# Female Genital Cutting and Hepatitis C Spread in Egypt

**DOI:** 10.1155/2013/617480

**Published:** 2013-05-07

**Authors:** Chris Kenyon, Jozefien Buyze, Ludwig Apers, Robert Colebunders

**Affiliations:** ^1^HIV/STD Unit, Institute of Tropical Medicine, Antwerp, Nationalestraat 155, 2000 Antwerp, Belgium; ^2^Division of Infectious Diseases and HIV Medicine, University of Cape Town, Anzio Road, Observatory 7700, South Africa; ^3^Department of Clinical Sciences, Institute of Tropical Medicine, Antwerp, Nationalestraat 155, 2000 Antwerp, Belgium

## Abstract

A recent analysis of Egypt's first nationally representative survey of hepatitis C virus (HCV) infection found female genital cutting (FGC) to be an independent risk factor for HCV infection for women in urban areas. We use the same dataset to extend this analysis. In an ecological analysis, we find a strong association between FGC and HCV prevalence (Pearson *R* ^2^—74%;  *P* < 0.0001). HCV prevalence is significantly higher if FGC is performed by a non-Doctor (15.4%) than a Doctor (4.2%; *P* < 0.001), and the calculated population attributable fraction of FGC for prevalent HCV seropositivity is high in women (79.8%).

## 1. Introduction

Why is Egypt's hepatitis C (HCV) epidemic so much more intense than elsewhere? Numerous lines of evidence point to the key role that the widespread use of parenteral antischistosomal therapy (PAT) played in this regard [1, 2]. However, question marks remain: numerous other countries have experienced similarly intense mass intravenous injections campaigns with inadequate attention to injection sterility without dramatic HCV epidemics [3, 4]. Furthermore, numerous contemporary studies have found that less than 20% of those testing HCV seropositive in Egypt have a history of PAT exposure [1, 2]. Although there is some evidence of a reduction in incidence [5], this is disputed [6], and HCV incidence has remained high [6]. Nonsterile medical and dental activities have been convincingly shown to be important in this regard [2, 6], but little or no evidence has been provided that these practices are more prevalent in Egypt than other middle-income countries. These considerations suggest that there may be other cofactors which may have been important in the generation of Egypt's uniquely high HCV prevalence. The results of a study to investigate the risk factors for prevalent HCV in Egypt using the country's first nationally representative sample found that female genital cutting (FGC) was associated with HCV on multivariate analysis but only in urban areas [1]. Since Egypt is one of a small number of countries where FGC is widespread this relationship merits more attention. In this paper we extend the investigation of the link between FGC and HCV. 

## 2. Materials and Methods

The Egyptian Demographic and Health Surveillance (EDHS), conducted in 2008, entailed a three-stage probability sample that provided a representative sample of 12,780 men and women aged 15–59. 11,126 (87.1%) of these agreed to provide blood for HCV testing. A third generation enzyme-linked immunosorbent assay was used to detect HCV antibodies (Adaltis EIAgen HCV Ab, Casalecchio di Reno, Italy). Full details of the survey and sampling strategy have been previously published [1, 7]. We further interrogated the relationship between FGC and HCV in four steps. 

Firstly, we performed a linear regression to analyze the ecological association between HCV and FGC prevalence by the six major areas that the EDHS was stratified by Urban and Frontier Governorates and Upper and Lower Egypt (each divided into rural and urban areas). This stratification is based on the fact that Egypt, in 2008, was divided into 26 governates. The four Urban Governorates (Cairo, Port Said, Alexandria, and Suez) have only urban populations. The other 22 governorates are divided into urban and rural areas. Nine of these governorates are in the Nile Delta (Lower Egypt) and eight are in the Nile Valley (Upper Egypt). The remaining five Frontier Governorates are located on the western and eastern boundaries of Egypt.

Secondly, we compared HCV prevalence according to who performed the circumcision. Thirdly, we described the changes in prevalence of FGC and HCV over time. FGC prevalence here is defined as the percentage of women who turn 15 on a particular year and who report having ever experienced FGC. This variable was defined in this way since the EDHS recruited women from age 15 and 99.6% of women who have ever experienced FGC have done so by age 15 [7]. The data for HCV prevalence trends is taken from the only longitudinal study of HCV prevalence from Egypt. This is a study of HCV seropositivity in 55,922 first time blood donors from the Mansoura region from 2000 to 2007 [5]. Fourthly, we calculate the population attributable fraction of FGS for HCV infection in women.

The statistical analyses were conducted in Stata 12.0 (College Station, TX; USA) using the survey (svy) methodology. All HCV prevalence rates and 95% confidence intervals (CI) were estimated using sampling weights.

## 3. Results

**Figure 1:**
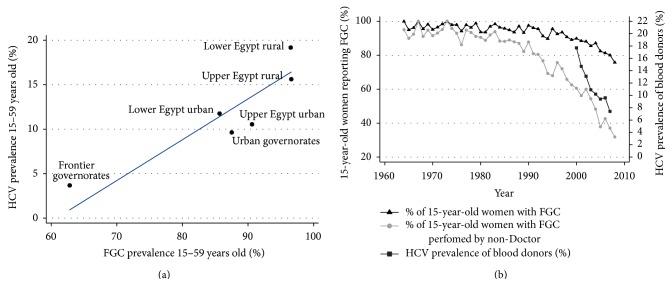
(a) Association between HCV prevalence and FGC prevalence in six areas in Egypt (Pearson *R* ^2^—74%; *P* < 0.0001). The line represents the least squares linear fit through the data. (b) Prevalence of female genital cutting (FGC) (from EDHS 2008) and HCV (from Ismail et al.) [5] by year.

In the ecological analysis (Figure 1(a)), we found a strong association between the prevalence rates of HCV and FGC at a regional level (Pearson *R* ^2^—74%; *P* < 0.0001). We repeated the same ecological analysis at the level of Egypt's 26 governates and found a weaker but still statistically significantly related correlation between HCV and FGC prevalence. Since the EDHS was designed to provide samples that were representative at the six regional levels but not at governate level we do not display these governate-level results. There was a significant difference in HCV prevalence according to who performed the FGC. This varied from 4.2% (95% CI 3.2–5.7) to 15.4% (95% CI 14.2–16.6) when a Doctor and non-Doctor, respectively, performed the procedure (*P* < 0.0001). 78.8% of all excisions were performed by informal health practitioners—most commonly Dayas (traditional birth attendants). 

As demonstrated in Figure 1(b), there has been a decline in the proportion of women reporting FGC. This decline is most marked in those reporting that they had FGC performed by a non-Doctor. HCV prevalence in first-time blood donors declined from 17.7% in 2000 to 7.4% in 2007 [5]. The declines in FGC prevalence by year predate the declines in HCV prevalence as demonstrated in Figure 1. The calculated population attributable fraction of FGC for HCV infection in women was 79.8%. 

## 4. Discussion

The analysis by Guerra et al. of the EDHS established that FGC was associated with HCV antibody positivity on multivariate logistic regression. This relationship was however only found to apply to women living in urban areas [1]. Why was this association found in urban but not rural areas? There are a number of possible explanations for this, one of which is that FGC is so prevalent in rural areas that there are too few women who are not excised in these areas to be able to demonstrate an effect of FGC on HCV prevalence. For example, in one of the few other studies to consider the impact of FGC on HCV in Egypt, no effect was found, but there was only 1 of 1989 individuals over 20 years old who was not excised [2]. In the EDHS only 4.0% (95% CI 3.2–5.0%) of women in rural areas, compared to 12.0% (95% CI 10.4–13.9%) in urban areas, reported not having had FGC performed. In this situation, where there may be insufficient heterogeneity in the exposure variable within particular regions, ecological analyses can be helpful. Our ecological analysis reveals that the highest prevalence rates for both HCV and FGC are in the rural areas and that there is a strong correlation between HCV and FGC overall. 

The association found between female HCV prevalence and having had FGC performed by a non-Doctor could be confounded by a number of other factors and thus the calculated population attributable fraction of FGC for HCV infection in women is likely an overestimate. Nevertheless the relationship between FGC and HCV merits further investigation. Other studies from Egypt have found a link between male circumcision being performed by traditional healers and prevalent HCV infection [8]. This issue is of relevance as the prohibitions on FGC legislated in 1995 mean that Doctors risk losing their license to practice if they perform FGC [7]. This could in turn lead to an increase in the proportion of FGC performed by non-Doctors. 

Although the decline in self-reported FGC, demonstrated in Figure 1(b), predates the decline in HCV prevalence noted in blood donors, this relationship needs to be treated with extreme caution. Firstly, the data from the blood donors was from the Mansoura area (Lower Egypt) and therefore not representative of Egypt. Secondly, no mention is made in the study of changes in the type of donors over this eight-year period. For example, if there was a trend in the donors being more likely to be younger or from urban areas (which are both associated with lower HCV prevalence rates) [1], this would have resulted in a decline of measured HCV prevalence with time. 

Inadequate sterility could be a risk factor for HCV at the time FGC is performed. This possibility is enhanced in a place like Egypt where traditional FGC occurred during a relatively short season, involving large numbers of persons being excised by a small number of operators [9, 10]. In addition the anatomical changes produced by FGC could promote subsequent female-to-male and male-to-female HCV transmission. A case-control study of primary infertility in Sudan found that cases were more likely to have undergone the most extensive form of FGC [11]. The evidence from Egypt is mixed. A case-control study of the determinants of infertility found that cases were more likely to have been excised by a traditional practitioner and more likely to have had more extensive forms of FGC [12]. A later study found no association between FGC and infertility [13]. A representative cross-sectional study from the Gambia found a strong association between prevalent FGC and Herpes Simplex Virus-2 infection (OR 4.7, 95% CI 3.7–6.4) and a weaker association between FGC and bacterial vaginosis [14]. 

The epidemiological evidence presented here adds to that from the individual level analysis performed by Guerra et al. [1]. Considerable more work is however required to unravel the complex interactions between FGC, who performs it, the type of FGC performed, and the subsequent risks for HCV transmission and acquisition.
